# Child Maltreatment Experience among Primary School Children: A Large Scale Survey in Selangor State, Malaysia

**DOI:** 10.1371/journal.pone.0119449

**Published:** 2015-03-18

**Authors:** Ayesha Ahmed, Choo Wan-Yuen, Mary Joseph Marret, Cheah Guat-Sim, Sajaratulnisah Othman, Karuthan Chinna

**Affiliations:** 1 Department of Social and Preventive Medicine, Faculty of Medicine University of Malaya, Kuala Lumpur, Malaysia; 2 Julius Centre University of Malaya (Centre for Clinical Epidemiology and Evidence Based Medicine), University of Malaya, Kuala Lumpur, Malaysia; 3 Department of Paediatrics, Faculty of Medicine, University of Malaya, Kuala Lumpur, Malaysia; 4 Paediatric Institute, General Hospital Kuala Lumpur, Kuala Lumpur, Malaysia; 5 Department of Primary Care Medicine, Faculty of Medicine, University of Malaya, Kuala Lumpur, Malaysia; Medical University of Vienna, AUSTRIA

## Abstract

Official reports of child maltreatment in Malaysia have persistently increased throughout the last decade. However there is a lack of population surveys evaluating the actual burden of child maltreatment, its correlates and its consequences in the country. This cross sectional study employed 2 stage stratified cluster random sampling of public primary schools, to survey 3509 ten to twelve year old school children in Selangor state. It aimed to estimate the prevalence of parental physical and emotional maltreatment, parental neglect and teacher- inflicted physical maltreatment. It further aimed to examine the associations between child maltreatment and important socio-demographic factors; family functioning and symptoms of depression among children. Logistic regression on weighted samples was used to extend results to a population level. Three quarters of 10–12 year olds reported at least one form of maltreatment, with parental physical maltreatment being most common. Males had higher odds of maltreatment in general except for emotional maltreatment. Ethnicity and parental conflict were key factors associated with maltreatment. The study contributes important evidence towards improving public health interventions for child maltreatment prevention in the country.

## Introduction

Child maltreatment is now recognised as a significant health and social problem in many nations. There is well established empirical evidence to show a ‘graded’ relationship between the number of unfavourable childhood maltreatment exposures and the occurrence of depression, poor academic performance and even suicidal ideation among youth [1, 2]. More recently, child maltreatment has also been linked to adult health risks such as alcoholism, smoking, having multiple sexual partners and sexually transmitted infections; as well as non-communicable diseases such as cancer, heart and liver diseases and obesity [3, 4].

Although it has been established that children are abused in a number of settings across the globe, most research into child maltreatment has been carried out in Western nations. While emerging evidence indicates that males may be more likely than females to experience harsh physical maltreatment in certain countries [5], data on gender differences in emotional maltreatment and neglect appears to be mixed [1, 6, 7]. Also, research from high income nations suggests that children experience substantially higher rates of maltreatment when living in a family with a single parent, a step-parent or parents who have substance abuse problems [6, 8]. These risk factors may appear to be less significant in some Asian cultures where cohabiting, divorce, or single parenthood is still relatively low [9]. For example, Choo et al (2011) [9] found no association between rates of parental divorce, single parents or step-parenting, or (apparent) parental drug use with different types of maltreatment or multiple victimization; while Ma et al (2011) found similar results where family structure had no influence on physical maltreatment [10]. Indeed, as research progresses, contrasts in maltreatment risk profiles are being observed between Western and Asian regions [11]. There is growing international interest in developing evidence based prevention programs, guided by empirical data on the extent of maltreatment and its risk factors in different populations [12]. It is therefore necessary to determine similar and/or differential risks, which would contribute towards understanding the factors influencing maltreatment, enabling the identification and targeting of appropriate and specific preventative measures.

In Malaysia, data on child maltreatment (more widely known as child abuse and neglect) is formally collected by the Department of Social Welfare, local Hospitals and the Royal Malaysian Police. It shows that over the years, reports of all kinds of abuse have increased, with the total number of cases tripling between 2001 and 2010, to more than 3000 per year [13]. Studies elsewhere have however found official estimates to be very conservative when compared to self-reported prevalence, picking up only the tip of the ice-berg [6]. Unfortunately, only a limited number of community/school surveys have been carried out in Malaysia [9, 14] to determine the actual public health burden of child maltreatment, and none so far among children of primary school age. The present study was carried out to estimate the prevalence of child maltreatment (parental physical maltreatment, emotional maltreatment and neglect as well as teacher-inflicted physical maltreatment) and cumulative maltreatment among primary school-going children in Selangor, Malaysia. It further aimed to examine demographic and social factors (gender; ethnicity; rural/urban settings; domestic violence) associated with child maltreatment among respondents, and to describe the psychological well-being of respondents.

## Methods

This cross-sectional survey was carried out in 2011 among primary school children in Selangor, which is Malaysia’s most populous state, with the highest reported number of child abuse and neglect cases in the country at that time [13]. Participants consisted of Year 5 students belonging to both rural and urban public primary schools. Public schools are thought to provide a better representation of the local population as the majority of Malaysian children attend these institutions, despite the existence of a number of private schools.

Sample Size was estimated using the formula n = [Z^2^ *p*(1-p)*DEFF]/d^2^, where Z is the critical value for 95% Confidence interval (1.96). The expected proportion of maltreatment in the population, p, was estimated using the prevalence of the least common type of maltreatment found in a similar cross sectional study carried out among older Malaysian adolescents [9] and taken as 20%. A Design Effect (DEFF) of 3 was considered appropriate for cluster sampling of schools. The absolute precision of the study was desired to be 2.5%. This yielded a minimum sample size of 2950.

The sampling frame of 641 rural and urban primary schools was provided by the Department of Education, Selangor. Two-stage stratified cluster random sampling was carried out to select 17 public primary schools (10 urban and 7 rural). These included 10 national schools where the main medium of instruction was Malay, and vernacular schools where the main medium of instruction was Chinese (4 schools) or Tamil (3 schools). The predominant ethnicity of the teacher and student population within each school corresponded to the medium of instruction. The total number of Year 5 classes in each school ranged from a minimum of 3 to a maximum of 11, with an average class size of 40 pupils. All Year 5 students in each selected school were invited to participate and it was hoped that this would reduce the stigma associated with participating in research of sensitive nature. This resulted in 3948 eligible students, of which 3509 proceeded to participate in the survey, giving a response rate of 88.9%.

The study was approved by the Medical Ethics Committee of the University Malaya Medical Centre (MEC Ref no 757.2) and Ministry of Education Malaysia. Written informed consent was obtained from school principals and parents were given the choice of having their child ‘opt out’ of participating. The ethics committee approved the consent procedure used in this study, and all the data was anonymized. The survey was conducted using anonymous self-administered questionnaires, completed in a single sitting, supervised by researchers and assistants. Students were assured of confidentiality and that participation was entirely voluntary. They were also provided with information on whom to contact, including details of the Child Helpline, if they wished to seek further advice.

Data was collected using a questionnaire prepared on the basis of previous literature review. A number of questions covering demographic, social and abuse variables were adapted from a list of pre-validated universally used instruments measuring child maltreatment including the International Society for the Prevention of Child Abuse and Neglect (ISPCAN) Child Abuse Screening Tool Children’s Version [15], the Child Exposure to Domestic Violence Scale [16] and others [17, 18]. These items were modified and simplified to suit the respondent’s cognitive ability, level of understanding and needs. The questionnaire was administered in Malay, Chinese or Tamil depending on student preference. A pilot study involving 298 students in 3 Selangor schools was initially carried out to ensure the clarity and cultural appropriateness of items in the questionnaire.

### Measures

The demographic construct included age, gender, ethnicity, geographical location and family characteristics (whether or not children lived at home, with or without their biological parents and the household composition). Socioeconomic status was measured using parental employment, classified based on government standards [19], along with a 10 point comfort score based on the availability of resources in the family for essential and non-essential items or activities (healthy food, clothes for school, holidays). Also measured was the degree of residential instability denoted by how often a student reported moving homes.

Items for parental characteristics included parental conflict (physical violence, threats or verbal abuse among parents), parental drinking and parental drug abuse; based on how frequently these were observed (scored as never = 1, sometimes = 2 and many times = 3). Parental conflict was deemed to be occurring if a child had a combined score of more than 3 for the three sub-items. Likewise, parental drinking and parental drug abuse were considered present if a child scored more than 1 on each item respectively. Parent child relationship was measured by asking children to describe how good (or bad) their relationship with each parent was and how often they ‘felt happy’ when in their company. Parental communication on safety was calculated by scoring how often children received advice on 6 items regarding personal protection and safety, from their parents. Scores were categorized into tertiles for ease of comparison.

For the purpose of the study, child maltreatment was measured by asking students to mark how often they suffered specific acts of maltreatment in 4 categories (S3 Table-parental physical maltreatment 5 items, S4 Table-parental emotional maltreatment 3 items, S5 Table-parental neglect 7 items and S6 Table-teacher inflicted physical maltreatment 7 items). Response categories included never, sometimes or many times (scored respectively as 1, 2 or 3), and positively worded statements were reverse coded. The reference time frame was broad, corresponding more to a life-time frequency rather than any recent period. Responses were summed up to calculate an aggregate score for each category and the mean score was used as a cut-off to demarcate the presence or absence of maltreatment. This method was screened for misclassification with regards to items that were judged as severe abuse according to local cultural norms (for example; hitting with stick or steel rod, burning and kicking the child hard). This was done by cross-tabulating individual items against the maltreatment outcome variable, and observing the number of cases that in fact had reported abuse but appeared under the ‘maltreatment absent’ category i.e. within a score below the mean. No misclassification was recorded. Cumulative maltreatment was defined as the presence of 2 or more categories of maltreatment.

The questionnaire also measured security of the home and school environment by asking how consistently a pupil felt safe at home and school; how regularly children felt happy at school, as well as whether or not children were willing to discuss problems at school with someone they trusted.

Psychological health of the participants was measured by the Malay, Chinese or Tamil version of the standardized Centre for Epidemiological Studies Depression Scale for Children (CES-DC) questionnaire. This consists of 20 items covering six main symptom areas and scored (0–3) depending on the frequency of their occurrence over the past week. The scale is best described as a screening instrument with higher scores indicating greater symptomatology [20].

### Analysis

All statistical analyses were performed using SPSS 16.0 for Windows. Weights were applied to the sample to adjust for the complex study design. Prevalence and socio-demographic characteristics were analysed using descriptive statistics. Chi square tests examined the relationship between qualitative variables like gender and ethnicity and the different categories of maltreatment. Complex samples logistic regression was used to test for associations between socio-demographic factors and different maltreatment categories. Multivariate analyses included all variables that were significant at p < 0.25 in univariate analyses, as well as others that were considered to be of important influence. Percentile scores were calculated for the CES-DC scale and multivariate analysis was also done for the associations between socio-demographic variables as well as maltreatment categories and obtaining a CES-DC score greater than the 90^th^ percentile. The latter was taken as a conservative cut off because it was less likely to include false positive symptoms of depression, as compared to lower scores. Seven different models were constructed and adjusted odd ratios (AOR) and 95% confidence intervals (CI) were presented where appropriate.

## Results

### Sample Characteristics

A total of 3509 students between 10 to 12 years old answered the questionnaire. In the sample 51.8% of the respondents were male and 56% were from an urban location. In terms of ethnicity, 45% were Malay, 36% Chinese and 19% Indian. Almost all (99.5%) of the children in the study were living at home, most of them (89.2%) with both biological parents. The family size of almost half (47.3%) of the respondents was between 6 to 8 persons.

Parental conflict was reported by 13.5% of the respondents, parental drinking by 8.9% and parental drug abuse by 1.7% (S1 Table). Fewer fathers were unemployed (4.7%) in comparison to 46.3% of the students whose mother was not working. Most of the children stated that they had a good or an excellent relationship with their father (95.5%) and mother (97.5%). Slightly more pupils were ‘happy most of the time’ when in the company of their mother (78%) than that of their father (69.7%). About 5% of the pupils recounted that they had moved house many times, compared to 48.6% who said that they had never moved house (S2 Table). Almost 80% of the respondents stated that they ‘always’ felt safe at home while about 50% felt that their school was ‘always’ safe. Similarly, when asked how often students felt happy while attending school, almost half the pupils students stated they were happy ‘most of the time’. More than 90% said they would discuss a problem occurring at school with someone they trusted (parent, teacher, friends).

### Prevalence of Child Maltreatment

Table 1 shows that for both genders, parental physical maltreatment was most common followed by parental neglect and parental emotional maltreatment. More than one quarter of all pupils reported having been physically maltreated by their teacher. Three quarters of ten to twelve year olds in Selangor were found to have experienced one or more forms of child maltreatment, while 43% were found to have suffered at least 2 forms. A minority of 2.6% reported all 4 types of child maltreatment

**Table 1:** Prevalence of specific type of maltreatment, cumulative abuse and range of CES-DC score.

Type of Maltreatment	Percentage of Respondents			Percentage in Population# (95% CI)		
	Male	Female	Total	Male	Female	Total
Parental Physical Abuse	54.5	42.8	48.9	60.1 (57.1–63.0)	44.7 (41.6–47.8)	52.7 (50.5–54.8)
Parental Emotional Abuse	25.6	20.3	23	23.0 (20.6–25.5)	18.0 (15.8–20.3)	20.5 (18.9–22.2)
Parental Neglect	45.1	32.2	38.9	44.2 (41.1–47.3)	29.2 (26.5–32.0)	37.0 (35.0–39.2)
Teacher Inflicted Physical Abuse	34.7	16.2	25.8	38.5 (35.4–41.8)	19.4 (16.7–22.4)	29.2 (27.1–31.5)
Cumulative Categories of Abuse						
None	18	32.8	25.3	16.2 (14.3–18.5)	33.1 (30.1–36.3)	24.5 (22.6–26.5)
1	31.1	35.7	33.2	29.7 (26.8–32.7)	35.1 (32.1–38.2)	32.3 (30.2–34.5)
2	29.9	21.1	25.7	32.0 (28.9–35.2)	21.0 (18.4–23.8)	26.5 (24.5–28.7)
3	17	8.9	13.1	18.1(15.6–20.9)	9.6 (7.7–11.7)	14.1 (12.5–15.8)
4	3.9	1.4	2.7	4.0 (2.9–5.5)	1.3 (0.8–2.1)	2.6 (2.0–3.5)
CES-DC Score						
≤15	41.2	40.7	40.9	41.9 (38.7–45.3)	41.1 (37.9–44.3)	41.5 (39.2–43.8)
16–20	21.3	20.5	20.9	21.7 (19.0–24.6)	21.7 (18.9–24.8)	21.8 (19.8–23.9)
21–25	18.1	16.9	17.5	17.6 (15.3–20.3)	17.8 (15.3–20.6)	17.7 (15.9–19.6)
26–30	10.2	10.1	10.2	10.2 (8.4–12.3)	9.6 (7.8–11.6)	9.9 (8.6–11.3)
31–35	5.4	6.1	5.8	5.4 (4.0–7.2)	5.4 (4.2–6.8)	5.4 (4.4–6.5)
36–40	1.7	3.2	2.4	1.4 (0.9–2.2)	2.7 (1.9–3.7)	2.0 (1.5–2.6)
>40	2.1	2.5	2.2	1.8 (0.9–3.8)	1.7 (1.0–3.2)	1.8 (1.2–3.0)

#Weights have been applied to the sample to adjust for complex study design.

### Demographic and Social Factors associated with the different types of Maltreatment

Associations between socio-demographic factors and child maltreatment are shown in Table 2. Geographical location was not found to be significantly associated with child maltreatment except teacher-inflicted physical maltreatment, for which the odds in urban areas were significantly lower than rural areas (AOR 0.71 95%CI 0.56–0.91).

**Table 2:** Multivariate associations between demographic, parental & environmental factors and child maltreatment.

Variables	Parental Physical Abuse OR (95% CI)°	Parental Emotional Abuse OR (95% CI)°	Parental Neglect OR (95% CI)°	Teacher Inflicted Physical Abuse OR (95% CI)°	Abuse of 2 or more kinds OR (95% CI)°
**Urban** ^**1**^	0.88 (0.69–1.11)	0.90 (0.69–1.18)	0.96 (0.75–1.21)	0.71 (0.56–0.91)	0.87 (0.68–1.11)
**Ethnicity** ^**2**^					
Chinese	0.24 (0.18–0.33)	3.08 (2.14–4.44)	1.30 (0.97–1.75)	0.21 (0.15–0.29)	0.50 (0.36–0.68)
Indian	1.70 (1.23–2.37)	1.19 (0.80–1.78)	0.68 (0.49–0.95)	0.81 (0.58–1.12)	0.99 (0.71–1.37)
**Male** ^**3**^	1.90 (1.50–2.41)	1.26 (0.97–1.63)	1.79 (1.43–2.25)	2.89 (2.25–3.71)	2.59 (2.04–3.29)
**Parental Conflict** ^**4**^	3.48 (2.45–4.96)	2.28 (1.64–3.17)	1.49 (1.06–2.08)	2.02 (1.47–2.77)	2.74 (1.97–3.81)
**Parental Alcohol Abuse** ^**5**^	1.35 (0.91–2.01)	1.96 (1.35–2.86)	1.09 (0.74–1.62)	-	1.95 (1.29–2.93)
**Parental Drug Abuse** ^**6**^	1.87 (0.54–6.42)	1.20 (0.39–3.65)	1.61 (0.40–6.41)	-	2.37 (0.57–9.82)
**Family Structure** ^**7**^	0.84 (0.51–1.39)	1.05 (0.63–1.74)	1.42 (0.87–2.30)	-	1.02 (0.62–1.67)
**Father’s Occupational Status**					
Unemployed^8^ ^!^	-	-	1.93 (1.11–3.36)	-	-
**No. of people in household** ^**9**^					
6–8	1.15 (0.90–1.46)	1.13 (0.86–1.47)	0.86 (0.68–1.09)	-	1.11 (0.87–1.43)
≥9	1.12 (0.73–1.72)	1.08 (0.67–1.75)	0.95 (0.64–1.42)	-	1.06 (0.70–1.61)
**Move House** ^**10**^					
Sometimes	1.37 (1.06–1.76)	0.80 (0.60–1.06)	0.99 (0.76–1.27)	1.18 (0.91–1.53)	1.00 (0.77–1.30)
Many times	1.52 (0.78–2.93)	0.49 (0.25–0.97)	1.20 (0.71–2.03)	3.00 (1.76–5.12)	1.91 (1.03–3.55)
**Happy with Father** ^**11**^					
Sometimes	1.43 (1.01–2.03)	1.41 (0.99–2.00)	1.57 (1.18–2.09)	-	2.02 (1.48–2.76)
Never/No contact	1.78 (0.81–3.92)	1.34 (0.70–2.56)	0.72 (0.35–1.48)	-	1.13 (0.54–2.36)
**Happy with Mother** ^**12**^					
Sometimes	1.61 (1.11–2.35)	1.80 (1.22–2.65)	1.27 (0.93–1.72)	-	1.66 (1.18–2.33)
Never/No contact	1.97 (0.85–4.59)	3.47 (1.75–6.89)	1.93 (0.93–4.03)	-	1.93 (0.86–4.34)
**Parental Communication** ^**13**^					
Mid Tertile	1.68 (1.25–2.26)	1.41 (1.02–1.96)	1.93 (1.46–2.54)	-	1.74 (1.30–2.33)
Lowest Tertile	1.42 (1.06–1.91)	1.21 (0.89–1.66)	2.77 (2.12–3.63)	-	2.04 (1.52–2.74)
**Not Safe at home** ^**14**^	1.98 (1.48–2.63)	1.57 (1.18–2.08)	1.44 (1.09–1.91)	-	1.79 (1.34–2.38)
**Comfort Score** ^**15**^					
8–9	1.09 (0.82–1.44)	0.65 (0.49–0.86)	0.61 (0.47–0.79)	-	0.80 (0.60–1.05)
10	0.74 (0.55–1.00)	0.58 (0.42–0.80)	0.63 (0.48–0.85)	-	0.69 (0.51–0.92)
**Happy at school** ^**16**^					
Sometimes	-	-	-	1.43(1.10–1.86)	-
Never	-	-	-	0.75 (0.50–1.11)	-

° Weights have been applied to the sample to adjust for complex study design. Odds ratios are adjusted for other variables shown and represent population measures.

Referent values for independent variables are:

^1^Rural,

^2^Malay,

^3^Female,

^4^No parental conflict,

^5^No alcohol abuse,

^6^No drug abuse,

^7^Both biological parents,

^8^Father employed,

^9^Five persons in household,

^10^Never moved house,

^11^Mostly happy with father,

^12^Mostly happy with mother,

^13^Highest tertile of parental communication,

^14^Always safe at home,

^15^Average and below comfort score,

^16^ Happy most of the times

^!^ Father’s Occupational status was found to be significant only for Parental Neglect. Mother’s occupational status was not found to be significant in univariate analysis and therefore not included in multivariate modelling.

Ethnicity had significant associations with all forms of child maltreatment, albeit with no consistent patterns. Chinese children were less likely and Indian children more likely to report parental physical maltreatment in comparison to Malay children. The odds of parental emotional maltreatment were 3 times higher among Chinese children while Indian children had significantly lower odds of parental neglect in contrast to their Malay counterparts. Additionally, Chinese students were less likely to report teacher-inflicted physical maltreatment, as well as cumulative maltreatment of 2 or more categories (OR 0.5 95%CI 0.36–0.68) compared to Malay pupils.

With regard to gender, the odds of child maltreatment including cumulative maltreatment were significantly higher in males relative to females, particularly for teacher-inflicted physical maltreatment (Table 2). The exception to this was parental emotional maltreatment where no significant gender association was demonstrated. Itemised prevalence of all categories of maltreatment reflect a similar gender pattern (Tables S3, S4, S5 and S6).

There was an association between parental conflict and individual maltreatment categories as well as the experience of multiple types of maltreatment. This association was particularly prominent among respondents who experienced significant parental physical maltreatment. Parental alcohol abuse was associated with nearly twice the odds of parental emotional maltreatment as well as multiple forms of maltreatment. However parental drug abuse which had a low prevalence in this sample population was not seen to be significantly associated with any form of child maltreatment.

Family structure and household occupancy were not found to be significant correlates of child maltreatment. Moving frequently increased the odds of teacher-inflicted maltreatment by three times but was not remarkably associated with any other forms of maltreatment. Difficulty in the relationship with either parent (less frequently happy) was associated with higher odds of multiple maltreatment. However maternal child relationship was also seen to be associated more broadly with individual categories of parental maltreatment, particularly emotional maltreatment.

Other important associations included significantly increasing odds of parental neglect as well as multiple forms of child maltreatment with deteriorating levels of parental communication about safety. Among students who reported not feeling safe at home, the odds of individual categories of parental maltreatment, physical maltreatment in particular, and multiple types of parental maltreatment were significantly higher.

The employment status of either parent was not found to be significantly associated with most categories of child maltreatment. However in children whose fathers were unemployed, the odds of parental neglect were 1.93 times higher (95%CI 1.11–3.36). When employment categories were explored in univariate analyses, there was an apparent inverse trend between child maltreatment and parental education as reflected by their employment skill level. Unfortunately a large number of missing data with regard to this particular measure precluded its use in multivariate models. However higher comfort scores, a proxy indicator of socioeconomic standing, were associated with significantly lower odds of parental emotional maltreatment and neglect. (Table 2)

### Symptoms of Depression

Almost 41% of the respondents scored 15 or less on the CES-DC scale (Table 1). The median score was 18 and the 75^th^ and 90^th^ percentile scores were 24 and 31 respectively. Multivariate analysis revealed that pupils experiencing any form of parental maltreatment, particularly parental emotional maltreatment, were also more likely to score >31. (Table 3) However, male students were found to have less than half the odds of obtaining a score greater than 31 when compared to their female counterparts. Furthermore, when compared to Malay pupils, Indian pupils were less likely to score with in the top 10% on the CES-DC scale (Table 3). Scoring >31 on the CES-DC was also noted to be strongly associated with how frequently a child felt happy at school in an expectedly negative manner.

**Table 3:** Multivariate associations between a CES-DC score greater than 31 and socio-demographic factors, and (A) maltreatment categories as well as (B) cumulative maltreatment.

Variables	Population Adjusted OR (95%CI)^@^	Population Adjusted OR (95%CI)^@^
**Region**		
Urban^1^	1.192 (0.79–1.81)	1.20 (0.80–1.82)
**Ethnicity** ^**2**^		
Chinese	1.608 (0.93–2.78)	1.85 (1.13–3.02)
Indian	0.365 (0.18–0.75)	0.34 (0.17–0.70)
**Gender** ^**3**^		
Male	0.439 (0.29–0.66)	0.41 (0.27–0.61)
**Parental Conflict** ^**4**^	1.417 (0.87–2.31)	1.45 (0.89–2.37)
**Parental Alcohol Abuse** ^**5**^	1.080 (0.65–1.80)	1.15 (0.69–1.92)
**Parental Drug Abuse** ^**6**^	1.074 (0.31–3.78)	1.03 (0.29–3.71)
**Family Structure** ^**7**^		
Others	1.531 (0.89–2.64)	1.39 (0.84–2.29)
**Move House** ^**8**^		
Sometimes	0.966 (0.62–1.50)	0.97 (0.62–1.52)
Many times	2.054 (0.92–4.60)	1.95 (0.92–4.12)
**Feeling not safe at home** ^**9**^	1.608 (1.08–2.39)	1.66 (1.12–2.45)
**Happy at school** ^**10**^		
Sometimes	2.373 (1.54–3.66)	2.37 (1.55–3.65)
Never	3.736 (1.85–7.53)	3.69 (1.82–7.49)
**Parental Physical Abuse** ^**11**^	1.828 (1.18–2.84)	-
**Parental Emotional Abuse** ^**12**^	2.194 (1.45–3.31)	-
**Parental Neglect** ^**13**^	1.898 (1.31–2.75)	-
**Teacher Inflicted Physical Abuse** ^**14**^	1.395 (0.88–2.21)	-
**Cumulative Categories of Abuse** ^**15**^		
**1**	-	1.90 (1.01–3.56)
**2**	-	2.56 (1.35–4.86)
**3**	-	4.47 (2.10–9.53)
**4**	-	21.55 (8.40–55.31)

^@^ Weights have been applied to the sample to adjust for complex study design. Odds ratios are adjusted for all other variables shown and represent population measures.

Referent values for independent variables are:

^1^Rural,

^2^Malay,

^3^Female,

^4^No parental conflict,

^5^No alcohol abuse,

^6^No drug abuse,

^7^Both biological parents,

^8^Never moved house,

^9^Always safe at home,

^10^Happy most of the times,

^11^No parental physical abuse,

^12^No parental emotional abuse,

^13^ No parental neglect,

^14^No teacher inflicted physical abuse,

^15^ No abuse of any kind

It is also worth mentioning that while parental conflict, parental drinking and parental drug abuse were significantly associated with a CES-DC score >31 in univariate analysis, adjustment in the multivariate model abolished this association. The analysis also shows that experiencing more than one category of maltreatment is significantly associated with greater depressive symptoms in an incremental fashion.

## Discussion

This is the first survey describing the prevalence of child maltreatment in Malaysian children of primary school age, based on self-report. The report of having at least one past experience of child maltreatment by three quarters of the population of 10 to 12 eleven year old primary school children is almost 3 times as high as that recorded in recent population surveys conducted among similarly aged respondents in UK [1] and Canada [21]. Cumulative maltreatment (≥2 kinds of maltreatment) was noted at 43%, and was double that reported in the only other local survey of a similarly sampled group of older (mean age 16yrs) Malaysian students [9]. However the latter study among secondary school students measured a broader range of child maltreatment (including sexual maltreatment) and applied a more stringent cut off (75^th^ percentile as opposed to the mean used here) to define maltreatment.

The finding that parental physical maltreatment is the most common form of maltreatment in this study closely parallels the experience in neighbouring Singapore [22]. The prevalence of parental physical maltreatment (53%), approaches the upper end of the range of physical abuse (from as low as 0.4% for very severe abuse to as high as 66.3% for moderate physical abuse) recorded in convenience sample surveys from other countries in East Asia and the Pacific region [23]. The results support the observation that physical punishment is more common and widely tolerated in Asia [24].

The estimated prevalence of parental neglect in this study of 37% falls within the range (11–40%) reported in parental and children’s surveys from the East-Asia Pacific region [23] but is greater than 3 times the prevalence in some high income countries [6]. In western nations, neglect is the most common type of maltreatment observed both in officially substantiated cases of child abuse [6] as well as in most community surveys [1, 21]. Parental neglect also forms the greatest proportion of officially recorded cases of child abuse in Malaysia [13], but is only the second most common type of child maltreatment noted in this school study. One possible explanation is the underestimation of neglect, as children who are severely neglected may not be attending school in addition to other forms of neglect, and therefore remain unrepresented in this school-based study.

Approximately 1 in 5 children reported being emotionally maltreated in this current study. This turned out to be lower than the combined global prevalence (36.7%), and the prevalence for Asia (41.6%) estimated in a recent meta-analysis [7]. Other studies from the East Asia and Pacific region also reported much higher rates of emotional abuse, especially among similar convenience samples [23]. It is uncertain whether the lower prevalence reflects the reality or is a consequence of the more subjective questions used to measure emotional maltreatment that may be susceptible to greater individual variation in perception and response compared to measures used for physical maltreatment which related to more readily tangible experiences. Similar to the meta-analysis however [7], the prevalence was the same for both genders.

The finding that teacher-inflicted physical maltreatment was reported by 29% establishes that this continues to be practised despite a large body of evidence that questions its effectiveness at behaviour modification in the long-term [25]. Its association with the male gender and rural surroundings is quite consistent with studies from other Asian countries [26–28] as well as the West [29], which report a similar link across a wide range of prevalence. The significant and independent associations of physical maltreatment by teachers with both parental conflict and frequent residential moves may reflect difficulties of children coping with family instability and dysfunction. Frequent residential moves further pose the challenge of adjusting to differing expectations and rules, as well as new peer relationships in different school environments. The outcome of instability and dysfunction within the home environment may include reduced support for learning at home with consequent difficulty completing assignments as well as poor academic performance, inconsistent attendance as well as behavioural problems, all of which may be triggers for being punished at school. Recognising this as a factor which increases a child’s vulnerability is important for planning interventions to provide better support for this subset of students. Results of a separate analysis reveal that the odds of being physically maltreated by teachers at school are two times higher for children who are also physically maltreated at home. This may be because children who receive corporal punishment at home are more likely to manifest behavioural problems and aggression at school which may make them targets of physical punishment, thus reinforcing their enacting of violence as well as other forms of undesirable behaviour [29]. The practice of physical maltreatment at school also hinders learning through its detrimental effects on open communication and building of self-confidence in students who experience this as well as those who witness it [29]. As more nations have opted for alternative healthier disciplinary practices in the light of such evidence, it is hoped that Malaysia will progress beyond the current situation where the use of physical disciplinary force by teachers is officially sanctioned, albeit with some restrictions [30, 31]. To determine whether associations between teacher inflicted physical maltreatment and the related socio–demographic factors were influenced by the specific school type a child attended, a sensitivity analysis was carried out to adjust for school type. The analysis revealed no remarkable change to any of the respective odds ratios. Chinese pupils continued to have lower odds, perhaps resulting from a difference in teaching and disciplinary styles in Chinese-medium schools.

The study has highlighted parental conflict as a significant correlate of all forms of parental maltreatment. This is in agreement with a number of studies conducted both in developed [32] and developing countries [33, 34]. The experiences may represent part of a broader spectrum of violence at home viz. stress associated with interpersonal violence precipitating child abuse. They may also be the result of overall dysfunction of families in conflict which lack structure and care leading to greater behaviour problems in children, in turn making them targets for further violence, at home and outside. It is vital to address this by enhancing parental counselling and education services.

Among other important social factors, the study links parental alcohol abuse with cumulative maltreatment, a finding that is consistent with a large body of research from Western nations like the US [35] as well as Eastern ones like South Korea [36]. It is notable that in this study, parental drinking is predominantly reported by children of Chinese and Indian ethnicities as opposed to Malay, and this is linked to the religious prohibition regarding consumption of alcohol among Muslim Malays. In contrast to other studies however the survey did not find any significant links between two other social variables thought to influence child maltreatment. Firstly, studies in Asian and African [5], and some Western countries [37, 38] have concluded that overcrowding in the household is a predictor of child maltreatment. The results of this survey however do not include significantly higher odds of maltreatment with increasing household size. Secondly, previous studies in the US [39, 40] and in Asia [36, 41] provide evidence for the association between low socioeconomic status of parents and the odds of child maltreatment. In this study, the inability to establish a reliable measure of socioeconomic status in sufficient numbers precludes any definite conclusion regarding its association with maltreatment.

Depression in adolescence and beyond has been linked with child maltreatment in Malaysia [42] and abroad [43]. It is also believed that such internalising behaviour has its onset in childhood [6]. The greater symptomatology of depression associated with all the three parental child maltreatment categories in this study is therefore a cause for concern, and underscores the need for a formal evaluation and follow up of children experiencing all forms of maltreatment. The fact that parental conflict fails to show an independent association may indicate that its influence is perhaps indirectly exerted through the unfavourable home environment it creates and the consequent maltreatment behaviour that results. The progressively increasing odds of a higher CES-DC score with increasing number of maltreatment types has also been reported elsewhere [11]. As such this calls for greater efforts in the prevention of child maltreatment as well as better surveillance on maltreatment by social workers, school authorities and multidisciplinary teams, to make early detection of damaging health consequences possible.

In this study, associations with gender show male pupils to be more vulnerable to all maltreatment categories except parental emotional maltreatment. This is consistent with current child maltreatment research done abroad except for one important difference, that being the association of parental neglect with the female gender seen in other populations, which is not seen here [5]. Furthermore, the observation that male gender has lower odds of obtaining a higher CES-DC score, in spite of greater prevalence of most forms of child maltreatment is a somewhat unexpected result. It may be related to other factors that can contribute to depressive symptoms and which have not been measured here, for example sexual and peer abuse. Alternatively, the finding may be an indication that the manifestation of depression in the two genders is independent of maltreatment, meriting further analyses.

Interpretation of the results must be considered in the light of the following limitations. The lack of temporality in the collected data makes it difficult to ascertain the predictive nature of the studied child and parent variables. For example, children’s perception of whether or not they feel safe at home could either precede or be the result of child maltreatment. Secondly, the survey requires respondents to recall maltreatment events over the extended past, which may introduce a bias in reporting their occurrence. Furthermore, the survey has not studied sexual maltreatment which is acknowledged as a key contributor to the overall burden of child maltreatment. Due to the prevailing cultural sensitivities associated with discussing this subject with young children, this aspect was not included in this survey.

## Conclusion

This study has provided an insight into the substantial burden of child maltreatment in a vulnerable age group in Selangor, Malaysia and reveals that it is comparable to regional estimates. The analysis draws attention to differences in prevalence of individual categories and multiple types of maltreatment with respect to ethnicity and gender. It has also highlighted the influence of parental relationships and the home environment (particularly with respect to residential instability); on the occurrence of maltreatment, as well as on children’s psychological wellbeing. It thus builds a case for conducting surveys in the wider community of parents and children, that explore in detail characteristics like parental education and parenting styles. It further emphasizes the need for reviewing child maltreatment prevention programs in general and disciplinary practices in particular.

## Supporting Information

S1 Table Parental Characteristics and Parent-Child Relationship factors related to the sample (N = 3509). (DOCX)

S2 Table Factors in the Social Environmental related to the sample (N = 3509). (DOCX)

S3 Table Item by item prevalence of Physical Maltreatment by Parents in both genders. (DOCX)

S4 Table Item by item prevalence of Parental Emotional Maltreatment in both genders. (DOCX)

S5 Table Item by item prevalence of Parental Neglect in both genders. (DOCX)

S6 Table Item by item prevalence of Teacher Inflicted Physical Maltreatment in both genders. (DOCX)
