# Supplementary material for: Child Maltreatment Experience among Primary School Children: A Large Scale Survey in Selangor State, Malaysia
Source: PLoS One. 2015 Mar 18;10(3):e0119449. doi: 10.1371/journal.pone.0119449 (PMC4364765; doi:10.1371/journal.pone.0119449)
Supplement: S1 Table — (DOCX) [file pone.0119449.s001.docx]

**Table S1:** Parental Characteristics and Parent-Child Relationship factors related to the sample (N=3509)

| **Variable** | **Frequency (percentage)*** |
| --- | --- |
| ***Parental characteristics*** |  |
| **Father’s Occupation** |  |
| Employed | 3195 (95.3%) |
| Unemployed | 156 (4.7%) |
| **Occupational Group Father** |  |
| 0-Armed Forces | 22 (0.9%) |
| Skill level 1 | 67 (2.8%) |
| Skill level 2 | 1442 (59.6%) |
| Skill level 3 | 216 (8.9%) |
| Skill level 4 | 674 (27.8%) |
| **Mother’s Occupation** |  |
| Employed | 1829 (53.2%) |
| Unemployed | 1610 (46.8%) |
| **Occupational Group of Mother** |  |
| 0-Armed Forces | 2 (0.2%) |
| Skill level 1 | 49 (3.7%) |
| Skill level 2 | 615 (46.6%) |
| Skill level 3 | 81 (6.1%) |
| Skill level 4 | 573 (43.4%) |
| **Parental Conflict** |  |
| No | 3004 (86.5%) |
| Yes | 468 (13.5%) |
| **Parental Drinking** |  |
| No | 3160 (91.9%) |
| Yes | 310 (8.9%) |
| **Parental Drug Abuse** |  |
| No | 3402 (98.3%) |
| Yes | 59 (1.7%) |
| ***Parent-child relationships*** |  |
| **Relationship with Father** |  |
| Good/Excellent | 3215 (95.5%) |
| Bad/Very Bad/No contact | 74 (2.2%) |
| Don’t know | 78 (2.3%) |
| **Relationship with Mother** |  |
| Good/Excellent | 3371 (97.5%) |
| Bad/Very Bad/No contact | 44 (1.3%) |
| Don’t know | 42 (1.2%) |
| **Happy with Father** |  |
| Mostly | 2347 (69.7%) |
| Sometimes | 928 (27.6%) |
| Never/No contact | 92 (2.7%) |
| **Happy with Mother** |  |
| Mostly | 2688 (78.1%) |
| Sometimes | 682 (19.8%) |
| Never/No contact | 72 (2.1%) |

*Excludes missing values
