# Supplementary material for: Child Maltreatment Experience among Primary School Children: A Large Scale Survey in Selangor State, Malaysia
Source: PLoS One. 2015 Mar 18;10(3):e0119449. doi: 10.1371/journal.pone.0119449 (PMC4364765; doi:10.1371/journal.pone.0119449)
Supplement: S2 Table — (DOCX) [file pone.0119449.s002.docx]

Table S2: Factors in the Social Environmental related to the sample (N=3509)

| ***Environmental factors*** |  |
| --- | --- |
| **Move House** |  |
| Never | 1681 (48.6%) |
| Sometimes | 1347 (38.9%) |
| Many times | 165 (4.8%) |
| Don’t know | 267 (7.7%) |
| **Feel safe at home** |  |
| Always | 2700 (78.0%) |
| Sometimes/Never | 763 (22.0%) |
| **Feel safe at school** |  |
| Always | 1891 (55.3%) |
| Sometimes/Never | 1531 (44.7%) |
| **Happy at school** |  |
| Most of the times | 1710 (49.3%) |
| Sometimes | 1470 (42.4%) |
| Never | 287 (8.3%) |
| **Would discuss Problem at school** |  |
| With someone they trust | 3235 (93.1%) |
| No one | 241 (6.9%) |

*Excludes missing values
