# Supplementary material for: Child Maltreatment Experience among Primary School Children: A Large Scale Survey in Selangor State, Malaysia
Source: PLoS One. 2015 Mar 18;10(3):e0119449. doi: 10.1371/journal.pone.0119449 (PMC4364765; doi:10.1371/journal.pone.0119449)
Supplement: S3 Table — (DOCX) [file pone.0119449.s003.docx]

Table S3: Item by item prevalence of Physical Maltreatment by Parents in both genders

| Item | **Parental Physical Maltreatment** |  | **Percentage of Respondents** | | **Percentage in Population# (95% CI)** | |
| --- | --- | --- | --- | --- | --- | --- |
|  | *Have your parents ever…?* |  | Boys | Girls | Boys | Girls |
|  |  |  |  |  |  |  |
| 1. | Hit or slapped you when they are angry with you | Never | 51.3 | 60.3 | 46.4 (43.3-49.4) | 58.4 (55.3-61.4) |
|  |  | Sometimes | 41.0 | 34.7 | 44.4 (41.3-47.4) | 36.5 (33.5-39.5) |
|  |  | Many times | 7.7 | 4.9 | 9.3 (7.5-11.5) | 5.1 (4.0-6.5) |
|  |  |  |  |  |  |  |
| 2. | Hit you with a hard object such as a stick or steel rod | Never | 87.9 | 92.6 | 87.5 (85.5-89.3) | 92.4 (90.6-93.8) |
|  |  | Sometimes | 9.4 | 5.6 | 9.8 (8.2-11.7) | 5.2 (4.1-6.6) |
|  |  | Many times | 2.7 | 1.8 | 2.7 (1.9-3.7) | 2.4 (1.5-3.8) |
|  |  |  |  |  |  |  |
| 3. | Burnt you with a cigar, iron or other hot materials | Never | 95.9 | 97.7 | 95.2 (93.7-96.4) | 97.2 (95.8-98.2) |
|  |  | Sometimes | 2.6 | 1.6 | 3.1 (2.1-4.5) | 2.0 (1.2-3.4) |
|  |  | Many times | 1.5 | 0.7 | 1.7 (1.1-2.6) | 0.8 (0.4-1.5) |
|  |  |  |  |  |  |  |
| 4. | Tied you with a belt, to a tree or on a chair | Never | 96.6 | 97.9 | 96.5 (95.3-97.3) | 97.4 (96.0-98.3) |
|  |  | Sometimes | 2.3 | 1.4 | 2.3 (1.6-3.2) | 1.9 (1.1-3.2) |
|  |  | Many times | 1.1 | 0.7 | 1.3 (0.8-2.1) | 0.7 (0.4-1.5) |
|  |  |  |  |  |  |  |
| 5. | Kicked you hard | Never | 91.3 | 95.4 | 91.2 (89.3-92.7) | 94.9 (93.2-96.2) |
|  |  | Sometimes | 5.9 | 3.5 | 5.6 (4.4-7.0) | 3.5 (2.5-4.7) |
|  |  | Many times | 2.8 | 1.1 | 3.3 (2.3-4.7) | 1.6 (0.9-3.0) |

#Weights have been applied to the sample to adjust for complex study design
