# Supplementary material for: Child Maltreatment Experience among Primary School Children: A Large Scale Survey in Selangor State, Malaysia
Source: PLoS One. 2015 Mar 18;10(3):e0119449. doi: 10.1371/journal.pone.0119449 (PMC4364765; doi:10.1371/journal.pone.0119449)
Supplement: S4 Table — (DOCX) [file pone.0119449.s004.docx]

Table S4: Item by item prevalence of Parental Emotional Maltreatment in both genders

| Item | **Parental Emotional Maltreatment** |  | **Percentage of Respondents** | | **Percentage in Population# (95% CI)** | |
| --- | --- | --- | --- | --- | --- | --- |
|  | *Have your parents ever…?* |  | Boys | Girls | Boys | Girls |
|  |  |  |  |  |  |  |
| 1. | Scolded you by calling ‘stupid’, ‘useless’ or ‘anak sial*’ | Never | 73.6 | 78.2 | 76.5 (74.1-78.9) | 81.2 (79.0-83.3) |
|  |  | Sometimes | 20.6 | 18.4 | 18.5 (16.4-20.8) | 15.5 (13.7-17.5) |
|  |  | Many times | 5.8 | 3.4 | 4.9 (4.0-6.1) | 3.3 (2.3-4.7) |
|  |  |  |  |  |  |  |
| 2. | Hugged you because they love you@ | Never | 20.1 | 16.8 | 16.6 (14.7-18.7) | 14.6 (12.8-16.7) |
|  |  | Sometimes | 32.7 | 29.2 | 33.1 (30.2-36.1) | 26.0 (23.5-28.6) |
|  |  | Many times | 47.2 | 54.0 | 50.3 (47.2-53.4) | 59.4 (56.4-62.3) |
|  |  |  |  |  |  |  |
| 3. | Loved your brother(s)/sister(s) more than you | Never | 46.9 | 50.3 | 46.8 (43.7-49.8) | 50.0 (46.9-53.1) |
|  |  | Sometimes | 33.5 | 33.4 | 31.5 (28.8-34.4) | 33.6 (30.7-36.7) |
|  |  | Many times | 19.7 | 16.3 | 21.7 (19.1-24.5) | 16.4 (14.2-18.8) |

^#^Weights have been applied to the sample to adjust for complex study design. *Derogatory term cursing child as ill-fated/unlucky ^@^ Reverse coding used for item
