# Supplementary material for: Child Maltreatment Experience among Primary School Children: A Large Scale Survey in Selangor State, Malaysia
Source: PLoS One. 2015 Mar 18;10(3):e0119449. doi: 10.1371/journal.pone.0119449 (PMC4364765; doi:10.1371/journal.pone.0119449)
Supplement: S5 Table — (DOCX) [file pone.0119449.s005.docx]

Table S5: Item by item prevalence of Parental Neglect in both genders

| Item | **Parental Neglect** |  | **Percentage of Respondents** | | **Percentage in Population# (95% CI)** | |
| --- | --- | --- | --- | --- | --- | --- |
|  | *Have your parents ever…?* |  | Boys | Girls | Boys | Girls |
| 1. | Had a friendly chat with you^@^ | Never | 11.0 | 7.3 | 10.4 (8.7-12.4) | 6.1 (5.0-7.4) |
|  |  | Sometimes | 43.9 | 40.0 | 42.1 (39.1-45.1) | 37.9 (34.9-40.9) |
|  |  | Many times | 45.1 | 52.7 | 47.5 (44.5-50.6) | 56.1 (53.0-59.1) |
|  |  |  |  |  |  |  |
| 2. | Asked you about what you did in school or your school problems^@^ | Never | 14.4 | 11.8 | 13.4 (11.5-15.5) | 11.5 (9.6-13.7) |
|  |  | Sometimes | 45.1 | 41.4 | 45.9 (42.9-49.0) | 39.4 (36.5-42.5) |
|  |  | Many times | 40.5 | 47.1 | 40.6 (37.7-43.7) | 49.1 (46.0-52.2) |
|  |  |  |  |  |  |  |
| 3. | Taken you to hospital when you are sick^@^ | Never | 4.7 | 4.8 | 4.9 (3.8-6.4) | 4.5 (3.5-5.7) |
|  |  | Sometimes | 31.2 | 29.9 | 30.6 (27.9-33.5) | 27.6 (25.0-30.4) |
|  |  | Many times | 64.1 | 65.3 | 64.4 (61.5-67.3) | 68.0 (65.1-70.7)) |
|  |  |  |  |  |  |  |
| 4. | Left you alone in the house without any elders | Never | 44.5 | 53.0 | 45.3 (42.2-48.3) | 55.2 (52.1-58.3) |
|  |  | Sometimes | 43.5 | 38.0 | 42.6 (39.6-45.6) | 35.9 (33.0-38.9) |
|  |  | Many times | 12.0 | 8.9 | 12.1 (10.2-14.5) | 8.9 (7.3-10.8) |
|  |  |  |  |  |  |  |
| 5. | Allowed you to play alone in the playground | Never | 72.0 | 86.8 | 72.2 (69.4-74.8) | 86.8 (84.5-88.8) |
|  |  | Sometimes | 16.8 | 9.7 | 16.0 (13.9-18.2) | 9.3 (7.6-11.2) |
|  |  | Many times | 11.2 | 3.5 | 11.8 (10.0-14.0) | 4.0 (2.9-5.5) |
|  |  |  |  |  |  |  |
| 6. | Allowed you to walk alone to school | Never | 81.1 | 86.8 | 79.9 (77.1-82.5) | 87.3 (84.9-89.3) |
|  |  | Sometimes | 10.9 | 8.6 | 11.2 (9.3-13.5) | 7.7 (6.2-9.5) |
|  |  | Many times | 8.0 | 4.6 | 8.8 (7.0-11.0) | 5.0 (3.7—6.9) |
|  |  |  |  |  |  |  |
| 7. | Locked you inside the house without any elders | Never | 73.2 | 78.6 | 74.6 (71.9-77.1) | 81.5 (79.1-83.7) |
|  |  | Sometimes | 20.4 | 17.0 | 18.9 (16.7-21.3) | 14.8 (12.8-17.1) |
|  |  | Many times | 6.5 | 4.4 | 6.5 (5.1-8.3) | 3.7 (2.8-4.8) |

^#^Weights have been applied to the sample to adjust for complex study design. ^@^ Reverse coding used for item
