# Supplementary material for: Child Maltreatment Experience among Primary School Children: A Large Scale Survey in Selangor State, Malaysia
Source: PLoS One. 2015 Mar 18;10(3):e0119449. doi: 10.1371/journal.pone.0119449 (PMC4364765; doi:10.1371/journal.pone.0119449)
Supplement: S6 Table — (DOCX) [file pone.0119449.s006.docx]

Table S6: Item by item prevalence of Teacher Inflicted Physical Maltreatment in both genders

| Item | **Teacher Inflicted Physical Maltreatment** |  | **Percentage of Respondents** | | **Percentage in Population# (95% CI)** | |
| --- | --- | --- | --- | --- | --- | --- |
|  | *Have your teachers ever…?* |  | Boys | Girls | Boys | Girls |
| 1. | Hit or slap you when they are angry with you | Never | 64.3 | 76.5 | 57.9 (54.7-61.1) | 72.5 (69.4-75.4) |
|  |  | Sometimes | 31.4 | 22.1 | 37.4 (34.3-40.6) | 26.0 (23.2-29.1) |
|  |  | Many times | 4.3 | 1.4 | 4.7 (3.4-6.5) | 1.5 (0.9-2.3) |
|  |  |  |  |  |  |  |
| 2. | Hit you with a hard object like a stick or rod | Never | 91.9 | 95.5 | 92.0 (90.0-93.6) | 95.8 (94.5-96.8) |
|  |  | Sometimes | 6.3 | 4.0 | 6.3 (4.9-8.1) | 3.9 (2.9-5.1) |
|  |  | Many times | 1.8 | 0.5 | 1.7 (1.0-2.9) | 0.4 (0.2-0.8) |
|  |  |  |  |  |  |  |
| 3. | Caned you | Never | 22.8 | 29.5 | 24.3 (21.8-27.1) | 31.4 (28.5-34.4) |
|  |  | Sometimes | 64.7 | 66.3 | 62.8 (59.7-65.8) | 64.8 (61.7-67.7) |
|  |  | Many times | 12.5 | 4.2 | 12.9 (10.8-15.4) | 3.8 (2.8-5.2) |
|  |  |  |  |  |  |  |
| 4. | Kicked you hard | Never | 96.2 | 98.8 | 95.3 (93.8-96.5) | 98.1 (96.4-99.0) |
|  |  | Sometimes | 2.6 | 0.9 | 2.9 (2.1-4.0) | 1.6 (0.8-3.4) |
|  |  | Many times | 1.2 | 0.3 | 1.8 (1.0-3.1) | 0.3 (0.1-0.7) |
|  |  |  |  |  |  |  |
| 5. | Thrown books, or duster towards you | Never | 81.1 | 89.5 | 80.1 (77.3-82.6) | 87.6 (85.089.8) |
|  |  | Sometimes | 17.0 | 9.8 | 17.2 (14.9-19.8) | 11.4 (9.3-13.8) |
|  |  | Many times | 1.9 | 0.7 | 2.7 (1.6-4.5) | 1.0 (0.4-2.4) |
|  |  |  |  |  |  |  |
| 6. | Pulled your ears | Never | 31.9 | 60.1 | 33.3 (30.6-36.2) | 60.6 (57.4-63.6) |
|  |  | Sometimes | 60.4 | 37.4 | 58.5 (55.5-61.6) | 37.2 (34.2-40.4) |
|  |  | Many times | 7.7 | 2.5 | 8.1 (6.3-10.4) | 2.2 (1.5-3.1) |
|  |  |  |  |  |  |  |
| 7. | Pinched you | Never | 42.5 | 54.3 | 37.4 (34.6-40.2) | 49.3 (46.2-52.4) |
|  |  | Sometimes | 48.1 | 41.1 | 52.0 (48.9-55.0) | 46.1 (42.9-49.3) |
|  |  | Many times | 9.4 | 4.2 | 10.7 (8.6-13.1) | 4.6 (3.3-6.5) |

^#^Weights have been applied to the sample to adjust for complex study design
